# Strong Expression of Chemokine Receptor *CXCR*4 by Renal Cell Carcinoma Correlates with Advanced Disease

**DOI:** 10.1155/2008/626340

**Published:** 2008-12-30

**Authors:** Thomas C. Wehler, Claudine Graf, Stefan Biesterfeld, Walburgis Brenner, Jörg Schadt, Ines Gockel, Martin R. Berger, Joachim W. Thüroff, Peter R. Galle, Markus Moehler, Carl C. Schimanski

**Affiliations:** ^1^Third Department of Internal Medicine, Johannes Gutenberg University of Mainz, 55131 Mainz, Germany; ^2^Institute of Pathology, Johannes Gutenberg University of Mainz, Langenbeckstrasse 1, 55131 Mainz, Germany; ^3^Department of Urology, Johannes Gutenberg University of Mainz, Langenbeckstrasse 1, 55131 Mainz, Germany; ^4^Institute of Surgery, Johannes Gutenberg University of Mainz, Langenbeckstrasse 1, 55131 Mainz, Germany; ^5^Interdisciplinary Translational Oncological Laboratory (ITOL), Johannes Gutenberg University of Mainz, Langenbeckstrasse 1, 55131 Mainz, Germany; ^6^Unit of Toxicology and Chemotherapy, German Cancer Research Center, 69120 Heidelberg, Germany; ^7^First Department of Internal Medicine, Johannes Gutenberg University of Mainz, 55131 Mainz, Germany

## Abstract

Diverse chemokines and their receptors have been associated with tumor growth, tumor dissemination, and local immune escape. In different tumor entities, the level of chemokine receptor *CXCR*4 expression has been linked with tumor progression and decreased survival. The aim of this study was to evaluate the influence of *CXCR*4 expression on the progression of human renal cell carcinoma. *CXCR*4 expression of renal cell carcinoma was assessed by immunohistochemistry in 113 patients. Intensity of *CXCR*4 expression was correlated with both tumor and patient characteristics. Human renal cell carcinoma revealed variable intensities of *CXCR*4 expression. Strong *CXCR*4 expression of renal cell carcinoma was significantly associated with advanced T-status (*P* = .039), tumor dedifferentiation (*P* = .0005), and low hemoglobin (*P* = .039). In summary, strong *CXCR*4 expression was significantly associated with advanced dedifferentiated renal cell carcinoma.

## 1. Introduction

Renal cell carcinoma (RCC) is the sixth leading cause of cancer-related deaths in the Western world and comprises 2-3% of all newly diagnosed malignancies in adults. Among the different kidney neoplasms, it represents with 85% the largest fraction [1]. The age-adjusted incidence of RCC in Western nations is 5–12/100 000 in women or men, respectively, with a peak incidence in the 6th decade [2]. In practice, the only curable treatment is nephrectomy performed in early stages of the disease. However, about 30–50% of patients have already metastases at presentation, and approximately one third of the nephrectomized patients relapse and progress with metastatic disease. The preferential sites of metastasis are the regional lymph nodes, the lung, the liver, and the bones. Survival strongly depends on the tumor stage at presentation. The 5-year survival rate is approximately 50%, whereas the median survival in case of metastasis is less than one year [3–5]. The current standard treatment for metastasized RCC consists of the application of *IFN*-*α* and *IL*-2 [6]. Recently, phase II clinical trials using receptor-tyrosine kinase (*RTK*) inhibitors have shown more promising results and lead to approval by the Food and Drug Administration (FDA) and European Medicines Agency (EMEA) [2].

In vivo and in vitro results from different tumor entities suggest that organ-specific metastasis is partially governed by interactions of chemokine receptors on cancer cells and their corresponding chemokines expressed in target organs and the tumor bed. This process is considered to direct lymphatic and hematogenous spread and furthermore influences the sites of metastatic growth [7]. Chemokines and their respective G-protein-coupled receptors were initially described to mediate different pro- and anti-inflammatory responses [8]. In particular, the high expression of stromal cell derived factor 1*α* (*SDF*-1*α*), also known as *CXCL*12, by endothelial cells, biliary epithelial cells, bone marrow stromal cells, and lymph nodes results in a chemotactic gradient attracting *CXCR*4 expressing lymphocytes into those organs [9–15]. Most recently, *CXCR*4 has shifted into focus as it is the most common chemokine receptor expressed on cancer cells [16]. It was suggested to play an important role in tumor spread of colorectal, breast, and oral squamous cell carcinoma as all of them commonly metastasize to *SDF*-1*α* expressing organs [17–20]. Data obtained from in vitro as well as from murine in vivo models, analyzing the metastatic ability of *CXCR*4 in expressing cancer cells, underlined the key role of *CXCR*4 for tumor cell malignancy, as activation of *CXCR*4 by *SDF*-1*α* induced migration, invasion, and angiogenesis of cancer cells [21–23].

Therefore, we evaluated the expression of *CXCR*4 in renal cancer cell lines and specimens and correlated these results with the patients' clinicopathological parameters and survival.

## 2. Materials and Methods

### 2.1. Tissue Samples

Renal cell carcinoma samples were intraoperatively obtained from 113 patients with renal clear cell carcinoma who underwent surgery at the Department of Urology of the University of Mainz. The morphological classification of the carcinomas was conducted according to World Health Organization (WHO) specifications. Patients were followed up on a regular basis depending on the procedure performed.

### 2.2. Immunohistochemical Staining

The avidin-biotin-complex method (LSAB+ System-HRP Kit, Dako Cytomation, Hamburg, Germany) was used to detect the protein *CXCR*4 (anti-*CXCR*4, dilution 1 : 300; Capralogics Inc., Mass, USA). Formalin-fixed and paraffin-embedded tissues were deparaffinized and subsequently microwaved (600 W, 15 minutes) in citrate buffer (ph 6.0). After preincubation with hydrogen peroxide (LSAB+ System-HRP Kit, Dako Cytomation, Hamburg, Germany) and human AB plasma (Department of Transfusion, University of Mainz, Mainz, Germany), the primary antibodies were applied for one hour at room temperature. After incubation with the secondary antibody (LSAB+ System-HRP Kit, Dako Cytomation, Hamburg, Germany), the avidin-biotin complex was added and the enzyme activity was visualized with diaminobenzidine (LSAB+ System-HRP Kit, Dako Cytomation, Hamburg, Germany). Counterstaining was performed with haematoxylin (Roth, Karlsruhe, Germany). For negative controls only the secondary antibody was used. A negative control was performed for each sample (*N* = 113). For positive controls formalin-fixed and paraffin-embedded tissue samples of the human spleen were applied.

### 2.3. Evaluation of Immunostaining

Immunostaining was evaluated by three authors independently (T.C. Wehler, C. Graf, S. Biesterfeld), blinded to patient outcome and all clinicopathologic findings. The immunohistochemical staining was analyzed according to a scoring method as previously validated and described [17]. The tumors were classified into four groups based on the homogeneous staining intensity: 0, absent; 1, weak; 2, intermediate; 3, strong staining. In the case of heterogeneous staining within the same sample, the respective higher score was chosen, if more than 50% of cells revealed a higher staining intensity. If expression intensity was exactly in between two scores, the authors agreed on 0.5 point-steps. If evaluations did not agree, specimens were re-evaluated and reclassified according to the assessment given most frequently by the observers.

### 2.4. Statistics

The correlation of *CXCR*4 staining intensity with clinicopathological patterns was assessed with the *χ* ^2^ test and with the unpaired Student *t*-test (one/two sided), when appropriate. Survival rates were visualized applying Kaplan-Meier curves, and *P*-values were determined by log-rank test. *P* < .05 was considered significant and *P* < .001 highly significant in all statistical analyses.

## 3. Results

### 3.1. Tumor Characteristics and Patient Profiles

The selected group of patients represents the typical characteristics of renal cell carcinoma in industrialized countries.

### 3.2. Immunohistochemical Staining of *CXCR*4 in Renal Cell Carcinoma

**Figure 1:**
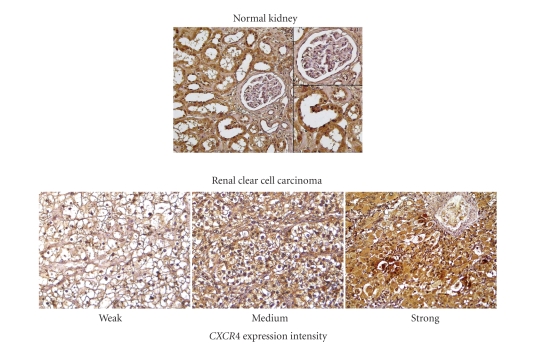
The figure depicts *CXCR*4 expression in healthy kidney and cancer samples. While glomeruli did not depict any *CXCR*4 expression, tubuli did reveal a medium-strong predominantly cytoplasmic *CXCR*4 expression. All cancer samples did reveal a cytoplasmatic expression of *CXCR*4 ranging from weak (34%) to medium (42%) and strong (24%).

The staining of normal human kidney tissue for *CXCR*4 revealed a cytoplasmatic expression and in only few specimens an additional weak membranous location of *CXCR*4 (see Figure 1). A nuclear staining of *CXCR*4 was not observed. In renal cell carcinoma, the respective expression rate for *CXCR*4 was 100% (113/113) and varied from weak (34%), intermediate (42%), to strong (24%). Negative controls of human renal cancer remained negative for all tissue samples (*N* = 113, not shown). Glomeruli did not reveal any *CXCR*4 expression and thus served as internal negative control. As internal positive control, splenic lymphocytes (strong *CXCR*4 expression) and tubuli cells (intermediate *CXCR*4 expression) were used. Similarly, inflammatory infiltrates in kidney tissue (data not shown) depicted a strong *CXCR*4 expression.

### 3.3. Relevance of *CXCR*4 Expression in Renal Cell Carcinoma

Strong *CXCR*4 expression significantly correlated with dedifferentiated (*P* = .0005) and progressed renal cell carcinoma, indicated by T-status (*P* = .039; see Table 1). Furthermore, strong *CXCR*4 expression revealed a significant association with low hemoglobin values (*P* = .039) and a nonsignificant trend towards increased thrombocytes (*P* = .089/*P* = .18, resp.). No correlation was seen for age, size, survival, or creatinine values.

## 4. Discussion

The expression of the chemokine receptor *CXCR*4 has been reported in various epithelial, mesenchymal, and hematopoietic tumors. In several entities, its expression was linked to tumor dissemination and poor prognosis [20, 24, 25]. *CXCR*4 expression can be increased as a result of intracellular second messengers such as calcium [26] and cyclic AMP [27, 28] by the inactivation of the tumor suppressor gene *p*53 and overexpression of *NF*κ*B* [29–31], by cytokines like *IL*-2, *IL*-10, or *TGF*-1*β* [26, 32] and by growth factors such as *VEGF* and *EGF* [33, 34]. In addition, Staller and colleagues could demonstrate that *CXCR*4 is a hypoxia inducible gene with a *HIF*-1*α* binding domain, and that its overexpression in clear-cell renal cell carcinoma is due to a loss-of-function of the von Hippel-Lindau (*VHL*) tumor suppressor protein, which under normoxic conditions directs *HIF*-1*α* to *ubiquitin*-mediated degradation [35]. Loss of *VHL* stabilizes *HIF*-1*α* leading to increased expression of hypoxia-response genes including *VEGFA*, *CXCR4,* its ligand *SDF*1*α*, and *HIF*-1*α* itself [36, 37]. They also reported a positive correlation between strong *CXCR*4 expression and poor tumor-specific survival independent of tumor stage and differentiation grade. The latter is in contrast to the results obtained in our study.

We analyzed the expression profile of *CXCR*4 in a series of human renal cell carcinoma cell lines and 113 patients' samples for which exact tumor staging and followup data were available and correlated the expression profile with clinicopathological data. The human renal cell carcinoma tumor samples that are analyzed revealed varying intensities of *CXCR*4 expression ranging from weak to strong, as previously described for pancreatic and colorectal cancer [38]. Interestingly, *CXCR*4 expression was downregulated in 34% and upregulated in 24% of renal cell carcinoma as compared to original tubuli cells. 42% of cancers revealed the identical expression intensity of *CXCR*4 as tubuli cells. A cytoplasmatic staining of *CXCR*4 was observed in all cancers, whereas fewer cases depicted an additional membranous localization of *CXCR*4. These observations are in line with a recently published study by Zagzag and coworkers [44]. Furthermore, it was reported that *CXCR*4 surface expression was higher in permanent cell lines than in primary tumor samples [39]. Noteworthy, an inducible translocation of *CXCR*4 from the cytoplasm to the membrane has been reported previously in [29]. In addition, at least in breast cancer cells, inhibited *CXCR*4 ubiquitination was described as another mechanism contributing to increased *CXCR*4 surface levels [40].

In our renal cell carcinoma patients, a strong *CXCR*4 expression was significantly associated as well with progressed cancer as indicated by the T-status as with dedifferentiation. Our results are furthermore in line with recent reports from our group and others, describing a similar effect of *CXCR*4 on disease progression in other tumor entities [17, 41]. Hence, our data suggest a relevant influence of *CXCR*4 on proliferation and differentiation of renal cell carcinoma with regard to the in vivo situation. This hypothesis is strengthened by observations in a murine model, where the metastatic capability of *CXCR*4-expressing RCC cells strongly correlated with *CXCR*4 protein level on cancer cells and the *SDF*-1*α* expression in the target organs [23]. Therefore, *CXCR*4-expressing cancer cells are certainly attracted to the typical “homing organs” such as lungs, bone marrow, liver, and lymph-nodes showing a high SDF-1*α* expression [13, 42]. A pathophysiological relevant fact worthwhile to be mentioned is that endothelial cells coexpress *SDF*-1*α* and *VCAM*-1, thus mediating tumor-cell/endothelial cell attachment. *CXCR*4 activation by *SDF*-1*α* induces **β*-integrin* expression, binding *VCAM*-1 on endothelial cell [43, 44]. Similar pathophysiological processes must be proposed for renal cell carcinoma dissemination.

Therefore, *CXCR*4 might be an interesting therapeutic target in a multimodal therapy of renal clear cell carcinoma.

## Figures and Tables

**Table 1:** Patient and tumor characteristics dependent on intensity of *CXCR*4 expression.

	*CXCR*4 expression			Statistics
	Weak	Medium	Strong	
Total number	**39** (34%)	**47** (42%)	**27** (24%)	
Average age (years)	63.8		66.3	*n.s.*
Gender				
* *Female	36 (42%)		8 (30%)	*n.s.*
* *Male	50 (58%)		19 (70%)	
Grading				
* *1/2	65 (78%)		11 (41%)	*P* = .0005
* *3/4	18 (22%)		15 (59%)	
T-status				
* *1/2	64 (76%)		15 (56%)	*P* = .039
* *3/4	20 (24%)		12 (44%)	
Average size (cm)	5.7		6.0	*n.s.*
Survival (months)	29.7		36.8	*n.s.*
Average creatinin (mg/dl)	1.11		1.07	*n.s.*
Average hemoglobin (g/dl)	14.32		13.25	*P* = .019*(1-sided)*
				*P* = .039*(2-sided)*
Average thrombocytes (/nl)	271		313	*P* = .089*(1-sided)*
				*P* = .18*(2-sided)*

## Abbreviations

CXCR4: Chemokine receptor 4
EMEA: European Medicines Agency
FDA: Food and Drug Administration
HIF: Hypoxia induced factor
IL: Interleukin
RCC: Renal cell carcinoma
RTK: Receptor-tyrosine kinases
SDF-1*α*: Stromal cell derived factor 1*α*
VHL: Von Hippel Lindau
WHO: World health organization.
